# Increasing on-target cleavage efficiency for CRISPR/Cas9-induced large fragment deletion in *Myxococcus xanthus*

**DOI:** 10.1186/s12934-017-0758-x

**Published:** 2017-08-16

**Authors:** Ying-jie Yang, Ye Wang, Zhi-feng Li, Ya Gong, Peng Zhang, Wen-chao Hu, Duo-hong Sheng, Yue-zhong Li

**Affiliations:** State Key Laboratory of Microbial Technology, School of Life Science, Shandong University, Jinan, 250100 People’s Republic of China

**Keywords:** CRISPR/Cas9, On-target cleavage efficiency, Spacer sequence, Free energy, Deletion of large genome fragments, Biosynthetic gene clusters for secondary metabolites, Epothilones, *Myxococcus xanthus*

## Abstract

**Background:**

The CRISPR/Cas9 system is a powerful tool for genome editing, in which the sgRNA binds and guides the Cas9 protein for the sequence-specific cleavage. The protocol is employable in different organisms, but is often limited by cell damage due to the endonuclease activity of the introduced Cas9 and the potential off-target DNA cleavage from incorrect guide by the 20 nt spacer.

**Results:**

In this study, after resolving some critical limits, we have established an efficient CRISPR/Cas9 system for the deletion of large genome fragments related to the biosynthesis of secondary metabolites in *Myxococcus xanthus* cells. We revealed that the high expression of a codon-optimized *cas9* gene in *M. xanthus* was cytotoxic, and developed a temporally high expression strategy to reduce the cell damage from high expressions of Cas9. We optimized the deletion protocol by using the tRNA–sgRNA–tRNA chimeric structure to ensure correct sgRNA sequence. We found that, in addition to the position-dependent nucleotide preference, the free energy of a 20 nt spacer was a key factor for the deletion efficiency.

**Conclusions:**

By using the developed protocol, we achieved the CRISPR/Cas9-induced deletion of large biosynthetic gene clusters for secondary metabolites in *M. xanthus* DK1622 and its epothilone-producing mutant. The findings and the proposals described in this paper were suggested to be workable in other organisms, for example, other Gram negative bacteria with high GC content.

**Electronic supplementary material:**

The online version of this article (doi:10.1186/s12934-017-0758-x) contains supplementary material, which is available to authorized users.

## Background

Clustered regularly interspaced short palindromic repeats (CRISPRs) play functions in prokaryotes as an acquired immune system, conferring host cells the resistance to exogenous genetic elements such as plasmids and phages [1–3]. Among the known CRISPR systems, the type II system utilizes a single CRISPR-associated (Cas) endonuclease protein, such as Cas9 from *Streptococcus thermophiles* [4], for sequence-directed DNA cleavage, guided by the CRISPR associated RNA (crRNA) for DNA targeting. The trans-activating RNA (tracrRNA) is a non-coding RNA that is able to form a RNA duplex with pre-crRNA for nuclease activity [5]. The two RNAs can be fused into a chimeric single guide RNA (sgRNA), which allows convenient and efficient delivery of the whole system for DNA cleavage targeting at the sequence containing a 20 nt protospacer and the NGG protospacer-adjacent motif (PAM) (5′-N20-NGG-3′; N indicates any base) [6]. The 20 nt guide RNA sequence can be easily replaced to retarget the Cas9 nuclease to a gene of choice. In recent years, the CRISPR/Cas9 system has been developed into a powerful genome-editing tool employable in different eukaryotes [7–12]. This genetic tool has also been harnessed for genome editing in bacterial species, including *Escherichia coli* [13], *Bacillus* [14], *Corynebacterium* [15], *Clostridium* [16, 17], *Lactobacillus* [18] and *Streptomyces* [19–21]. There are two major questions to be resolved for efficient applications of the CRISPR/Cas9 system. One is cell damage due to the endonuclease activity of introduced Cas9. The other is potential off-target DNA cleavage produced from incorrect guide by a selected 20 nt sequence, on which there have been reported many results, but often with paradoxical conclusions, for example, effects of the GC content on sgRNA activity [22, 23], the hairpin structures in sgRNA [10, 22] or the gRNA sequence features [24, 25] on cleavage efficiency.

The Gram-negative myxobacteria belong to the delta division of the Proteobacteria, and are well known for their multicellular social behaviors and the production abilities of diverse secondary metabolites [26–28]. The myxobacterial cells harbor large genomes [29–32], containing lots of genes related to the complex sociality and the biosynthesis of diverse secondary metabolites. The *Myxococcus xanthus* DK1622 is a model strain of the myxobacteria and has been developed as an efficient host for the expression of secondary metabolites from other difficult-to-handle myxobacteria, such as epothilone, haliangicin, disorazol [33–37]. The strain itself possesses 24 biosynthetic gene clusters for secondary metabolisms, occupying 8.6% of the 9.14-Mb circular genome, and is a potential producer of multiple secondary metabolites [38]. Up to now, six compounds (myxochelins, myxochromides, myxovirescin, DKxanthenes, myxalamids, and myxoprincomide) have been identified and correlated to their biosynthetic gene clusters in DK1622 [39–42]. According to the transcriptomic and proteomic analyses, the remaining unassigned pathways are also active under standard cultivation conditions [43]. It is industrially interesting to remodel the large genome of this model myxobacterium to a ‘microbial factory’ [44] for the production of bioactive secondary metabolites, which, however, is limited by lacking efficient genome editing protocols.

In this study, we established an efficient CRISPR/Cas9 system for the deletion of large genome fragments in *M. xanthus* DK1622. We developed a strategy for temporally high expression of Cas9 to decrease the cytotoxicity of this endonuclease in *M. xanthus*. We used a tRNA–sgRNA–tRNA strategy to ensure the production of correct sgRNA molecules. We found that, in addition to the position-dependent nucleotide preference, the free energy of a 20 nt spacer was a key factor for the deletion efficiency. By using the developed process, we successfully deleted large biosynthetic gene clusters for the production of secondary metabolites in *M. xanthus*. We assayed the deletion effects on yields of other secondary metabolites, including the heterologous epothilones.

## Results

### *M. xanthus* cells cannot tolerate highly-expressed Cas9 endonuclease

The 35.1% GC-content of *cas9* is greatly lower than that of the *M. xanthus* DK1622 genome (68.9%). To develop a CRISPR/Cas9 editing system in *M. xanthus*, we employed a codon-optimized *cas9* gene, which had been used in similarly high-GC-possessing *Streptomyces* species [19]. Cas9 is an endonuclease. Introducing the Cas9-encoding gene into *M. xanthus* might lead to cytotoxicity. To determine influences of Cas9 in *M. xanthus* cells, we inserted a copper-inducible promoter (P_cuoA_) [45] in front of the *cas9* gene to control its expression corresponding to the copper concentration. The tracrRNA and crRNA sequences were connected into a single sgRNA sequence (Additional file 1: Figure S1). The pET-based plasmids cannot replicate in *Myxococcus xanthus* cells. We constructed two pET-based suicide plasmids: pET28a–P_cuoA_-Cas9, which contains the *cas9* gene under the control of copper-inducible promoter P_cuoA_, and pET28a–P_cuoA_-Cas9–sgRNA, which has an additional T7A1-controlled sgRNA containing a 20 nt spacer targeting the myxochelin gene cluster (Additional file 2: Figure S2). The two plasmids were both integrated into the genome of DK1622 by a single crossover at the 3976002..3976945 complementary sequence site with the 944-bp P_cuoA_ promoter homologous arm, producing transformants DK-Cas9 and DK-Cas9–sgRNA, respectively, which were confirmed by PCR and sequencing.

**Fig. 1:**
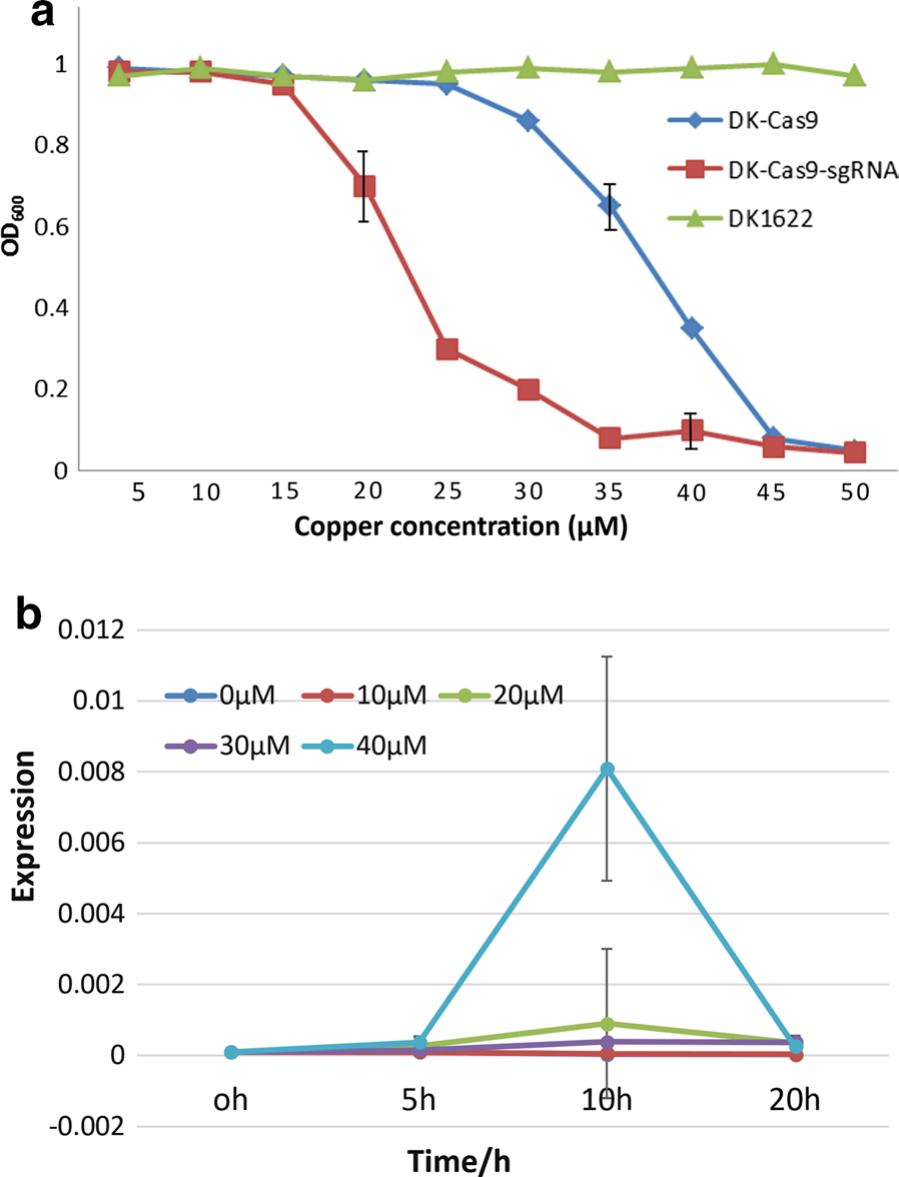
Effects of copper concentrations on the cellular growth of *M. xanthus* and the *cas9* gene expressions. **a** The growth abilities of DK1622 and the DK-Cas9 and DK-Cas9–sgRNA mutants in CTT medium containing different concentrations of copper. The cas9 gene is controlled by the P_cuoA_ promoter. Cells grown in the absence of copper were diluted to 0.05 OD_600_ value in the CTT medium containing the indicated copper concentration, which were then incubated at 30 °C for 24 h, and monitored the cell density. **b** Expressions of the *cas9* gene in DK-Cas9–sgRNA induced with different copper concentrations. At 0 h, the cells were treated with different copper concentrations in CTT medium. After 5 h of incubation, the cells were harvested by centrifugation, and further incubated in CTT medium. The *cas9* gene expressions in cell were assayed. Experiments were performed in triplicate

The DK-Cas9 and DK-Cas9–sgRNA mutants showed almost the same growth abilities as the wild type DK1622 in liquid CTT medium without the addition of copper. When different concentrations of copper were added into the medium, the growth abilities of these two mutants both became greatly weakened (Fig. 1a demonstrates the OD_600_ values of these cultures after 24 h of incubation in CTT liquid medium supplemented with different concentrations of copper). At the 12.5 μM copper concentration or lower, the OD_600_ values of the DK-Cas9 and DK-Cas9–sgRNA cultures were similar to that observed in DK1622. With the increase of copper concentration, the growth of DK-Cas9–sgRNA sharply decreased, whereas the DK-Cas9 mutant still had almost the same growth ability as DK1622 and markedly decreased at 35 μM of the copper concentration. The DK-Cas9–sgRNA and DK-Cas9 mutants showed almost no growth at the 35- and 45-μM copper concentrations, respectively. As the wild type strain DK1622 is able to tolerate up to 500 μM copper concentration [45], the growth inhibition of the mutant cells was suggested to be due to the toxicity of highly expressed *cas9* gene in *M. xanthus* cells, rather than the presence of copper in medium. We assayed the *cas9* expression changes in DK1622-Cas9–sgRNA after treating the cells with different copper concentrations for 5 h. The result showed that the *cas9* expressions were similar with low copper concentrations (≤30 μM), but greatly up-regulated at a higher copper concentration (40 μM) after additional 5-h incubation, then sharply decreased with the increase of incubation time (Fig. 1b). Thus, the introduced CRISPR/Cas9 system does not affect the growth of *M. xanthus* cells when the *cas9* expression was kept at low concentrations. The different inhibition tolerances of the two mutants on copper concentration suggested that the cell damage from the directed DNA cleavage by the sgRNA–aided Cas9 was more serious than the potential random cleavage by Cas9 (without sgRNA activation).

### Deletion of the myxovirescin gene cluster from the *M. xanthus* DK1622 genome

**Fig. 2:**
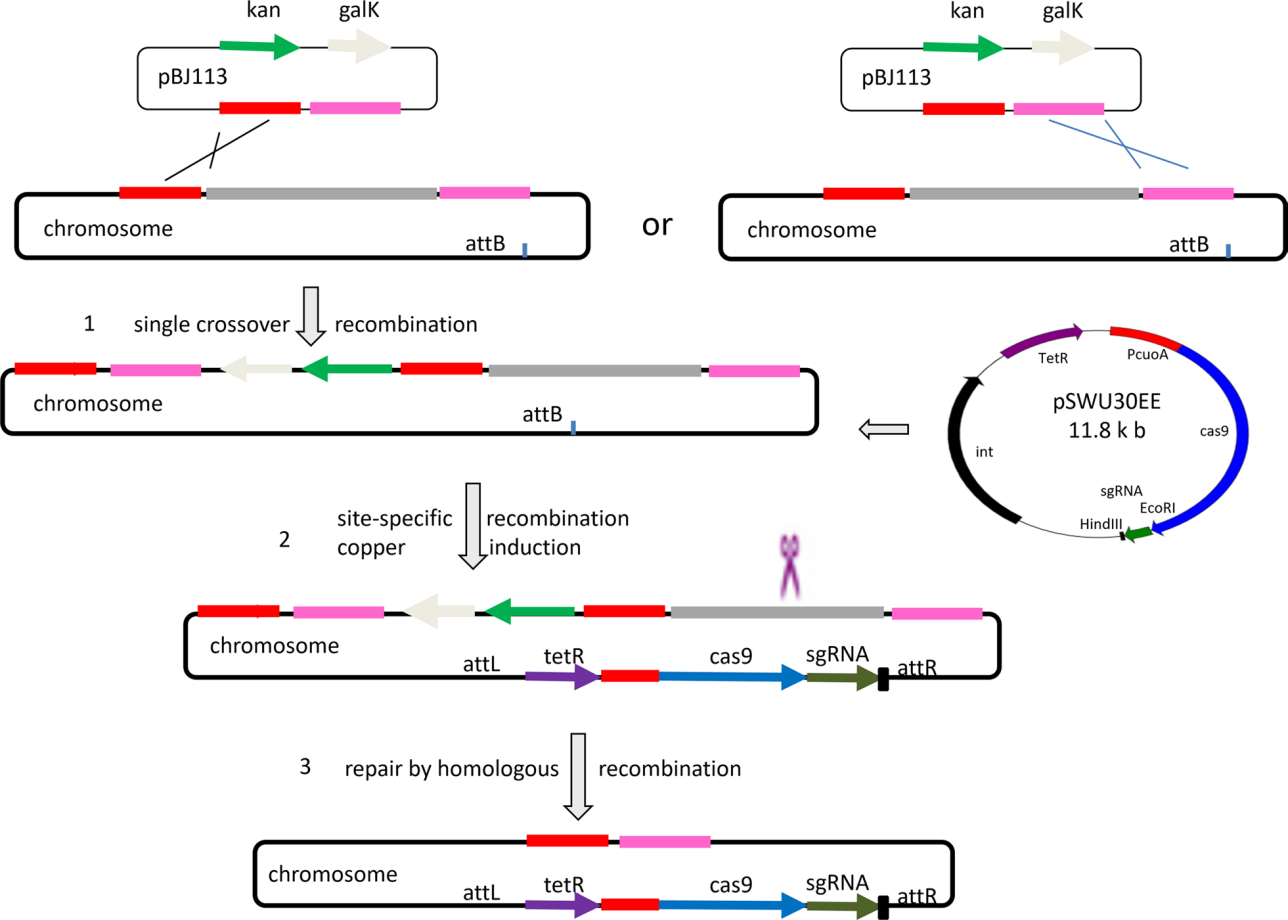
The diagrammatic sketch describing the progresses for the genome edition. Firstly, the suicide plasmid pBJ113 was integrated into the genome by a single crossover of homologous recombination at one of the two homologous arms constructed in the pBJ113 plasmid, which were designed at the places flanking the target gene cluster. Secondly, a CRISPR/Cas9 editing plasmid pSWU30EE was transferred into *M. xanthus* genome by site-specific integration. The pSWU30EE plasmid contained an inducible promoter-driven *cas9* gene and a constitutive promoter controlled sequence-specific sgRNA. Thirdly, the expressed Cas9 was guided by the sgRNA sequence to target at the complementary chromosomal site for cleaving, producing a double-strand break in the chromosome. The cleaved chromosome was repaired by the other homologous arm that was constructed in the pBJ113 plasmid in a homologous recombination repair mechanism. After a double crossover of homologous recombination, only the cells with the deletion of the target fragment were survived. According to the design, the recombination occurred at either of the two homologous arms, which had been constructed in the pBJ113 plasmid. During the deletion of the myxovirescin gene cluster in DK1622, we confirmed that integration of the pBJ113 plasmid into the genome of DK1622 occurred via the left arm by a single crossover of homologous recombination. After the integration of pSWU30EE and induction, the double-strand break was repaired by second crossover homologous recombination via the right homologous arm

**Fig. 3:**
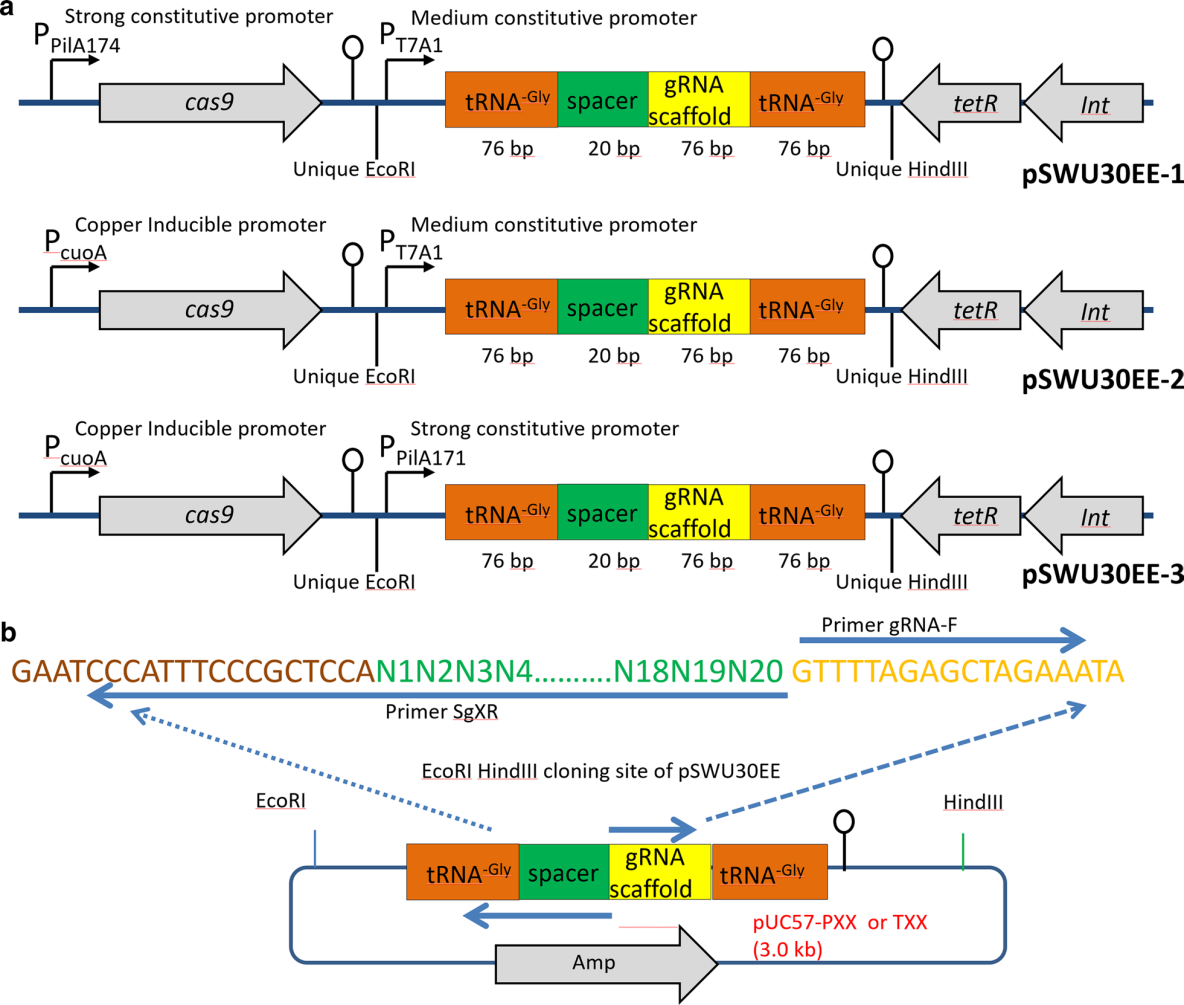
Vector design for Cas9/sgRNA mediated genome editing in *M. xanthus*. **a** The Cas9/sgRNA expression plasmids were constructed in vector pSWU30. The *cas9* gene was promoted by the constitutive promoter (P_pilA_) or the copper-inducible promoter (P_cuoA_), and the tRNA–sgRNA–tRNA chimeric gene was transcribed from the T7A1 promoter or the P_pilA_ promoter and terminated by T7 terminator. **b** Construction of sgRNA transcription cassette. The model cassette was artificially synthesized and cloned into pUC57. Two primers, general primer gRNA-F and custom primer SgXR, which contained specific 20 nt spacer, were used to produce new target site

Myxovirescin (TA) is a major kind of the secondary metabolites produced by *M. xanthus* DK1622, and the biosynthetic gene cluster is approximately 83 kb in size [38]. To delete the myxovirescin gene cluster, we employed the well-studied *attB*-site-specific integrating plasmid pSWU30 and suicide plasmid pBJ113 to integrate *cas9*–sgRNA and two homologous arms of the myxovirescin gene cluster into host chromosome, respectively (the process is demonstrated in Fig. 2). The spacer sequence is 11-2 (listed in Table 1), which is located in the downstream of the myxovirescin biosynthetic gene cluster. It was reported that inserting a tRNA sequence in front of sgRNA was able to improve the edition efficiency of the CRISPR/Cas9 system in plant cells [12]. We employed the strategy with a modification by adding a second tRNA gene following the sgRNA sequence, forming a tRNA–sgRNA–tRNA architecture to ensure the production of the sgRNA molecule (containing the 11-2 20-nt spacer and a 76-bp gRNA scaffold). The sgRNA expression cassette was approximately 400 bp, consisting of a promoter, a tRNA, a sgRNA, a second tRNA, and the terminator (details refer to Additional file 1: Figure S1, Additional file 2: Figure S2). The *cas9* gene was firstly cloned into pET28a by a double digestion with *Xba*I + *Eco*RI. The gene was under the control of the highly-efficient constitutive promoter P_pilA_, which was also substituted to the copper-inducible promoter P_cuoA_ (Fig. 3a). The sgRNA cassette was cloned into the intermediate plasmid by *Eco*RI + *Hin*dIII (Fig. 3b). The fragment containing the *cas9* gene and the sgRNA cassette was then obtained by a double digestion with *Xba*I + *Hin*dIII, and transferred to pSWU30E to form the pSWU30EE-1 and pSWU30EE-2 plasmids (Additional file 3: Figure S3), respectively.

**Table 1:** Information of the 5′-N20-NGG-3′ spacer candidates for the deletion of the biosynthetic gene clusters for myxovirescin and myxalamids

Name^a^	20 nt spacer sequence^b^	Hairpin	GC (%)	ΔG^c^
11-1	TGCAA*A*TCGGAGTAGAA*GCG* CGG	N	50	−39.6
11-2	GCCGACGCCGCCCTTGAT*GG* TGG	N	75	−49.0
11-3	GC*G*TG*A*G*G*AG*G*AAGCG*G*C*GG* TGG	N	75	−47.3
11-4	TCCTGTCCGAACCGCCC*GC*C CGG	N	75	−48.4
17-4	GACGAGGCACCTAGAGACAC TGG	N	60	−35.8
17-5	CCACTCATCCAGGACGCTAC TGG	N	60	−37.8
17-6	CGCGAGGCTGCGGCTGCCAC TGG	Y	80	−49.3
17-7	GACGCCCCATCCCCAACCAC TGG	N	70	−44.7
17-8	CGAGAGGTCGCTCTCCAAAC TGG	Y	60	−39.3
17-9	GGCGATGATGAGGAACCAAC TGG	N	55	−39.2
17-10	CCTGCTCCTGCGCCTTCCAC TGG	N	70	−44.0
Degenerate^d^	NVH-N2-BNH-N2-H-N5-HHAC TGG			

^a^The locations are referred to Fig. 4 for 11-1 to 11-4 and Fig. 5 for 17-4 to 17-10

^b^Disfavored nucleotides are shown in italics

^c^ΔG indicates free energy (kcals/mol). The calculation of free energy and prediction of hairpin in 20 nt spacer were performed by primer premier 5.00

^d^H: A/T/C, V: G/A/C, B: G/T/C, N: any nucleotide; the C-terminus TGG or CGG is the PAM motif

**Fig. 4:**
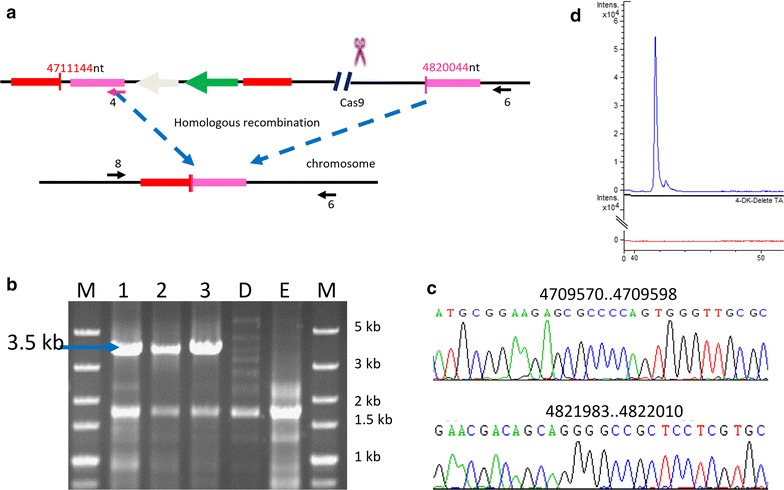
Large fragment deletion of the myxovirescin biosynthetic gene cluster in *M. xanthus* DK1622. **a** Schematic diagram for the deletion program. Left and right homologous arms are shown in *red* and *pink rectangles*, respectively. *Gray* and *green arrows* represent galK gene and kan^R^ gene, respectively. **b** Identification of mutant strains by agarose gel electrophoresis of PCR product. The PCR primers 8 and 6 (referred to the **a**) yielded the 3.5-kb positive band (indicated by *blue arrow*) in three independent mutants (*lanes 1–3*), but not in the negative controls of DK1622 (*lane E*) and DKpBJ11-tsg112 (*lane D*). **c** Chromatograms of the 3.5-kb PCR product DNA sequences, sequenced using the primers 8 and 6 as the sequencing primers. Since plasmid pBJ11 contains the nucleotide sequence crossing the deleted chromosomal region, we sequenced the flanked region at two ends of the 3.5-kb PCR product. **d** EIC of myxovirescin A ([*M*+H]^+^ *m/z* 624.44) from the DK1622 (*blue*) and mutant (*red*)

The pSWU30EE-1 plasmid, in which the *cas9* gene was driven by the constitutive promoter P_pilA_, produced no deletion mutant. This result was probably due to the lethality of high expressions of Cas9, which is consistent with the above results that the *M. xanthus* cells containing P_cuoA_-controlled *cas9* gene were unable to grow with high copper concentrations (Fig. 1). In contrast, transformation of pSWU30EE-2 (containing the P_cuoA_-controlled *cas9* gene) into the *M. xanthus* DK1622 produced kanamycin resistant colonies after the induction at the 25-μM copper concentration. Single colonies of the mutants were inoculated on CTT agar plate with or without kanamycin. From the total 30 colonies that appeared on the CTT plate without kanamycin, we obtained three strains, whose corresponding inoculates could not form colonies on the plate with kanamycin, suggesting they lost the kanamycin resistance. PCR amplification using the primers 8/6 annealing outside the 2 kb homology arms (Fig. 4a) produced the desired 3.5-kb band in these three Kan^−^ mutants, but none in the wild type strain (Fig. 4b), which suggested that the 83-kb myxovirescin gene cluster has been deleted in the three Kan^−^ mutants. The deletion was confirmed by sequencing (Fig. 4c). HPLC–MS assays also detected no myxovirescin in the mutant broth (Fig. 4d).

### Temporally high expression of the *cas9* gene improves the deletion efficiency

**Fig. 5:**
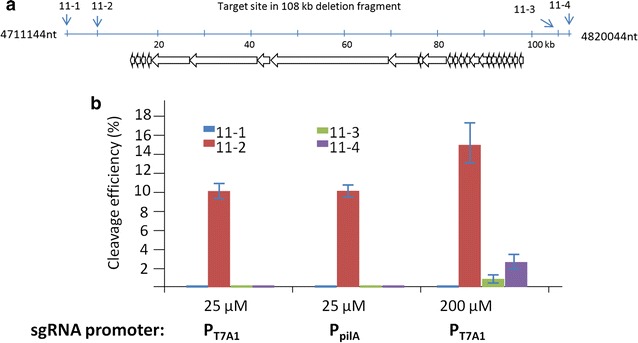
Comparison of the cleavage efficiency of gene cluster guided by four spacers under different conditions. **a** Four spacer positions (11-1 to 11-4) flanking the myxovirescin gene cluster were used as the sgRNA-targeting sites for deletion. The genes involved in the biosynthesis of myxovirescin are shown in the *arrows*. The positions of the guide sequences are indicated. **b** *Histograms* show the target cleavage efficiencies of the four sgRNAs including the 11-1 to 11-4 spacers, respectively, controlled with different promoters or different concentrations of copper ion. Experiments were performed in triplicate. *Error bars* indicate standard deviations

The above established CRISPR/Cas9 system was efficient in the deletion of the myxovirescin gene cluster in *M. xanthus* with an efficiency approaching 10% (3/30) of the bacterial population, but 100% of the three Kan^−^ mutants. Although the tRNA–sgRNA–tRNA strategy may improve the edition efficiency, the selected 20 nt spacer sequence was suggested to play a critical role for the on-target deletion efficiency. To evaluate effects of the 20 nt spacer, we further replaced the 11-2 spacer with 11-1, 11-3 or 11-4, which also flanked the myxovirescin gene cluster (Fig. 5a), to construct the series plasmids of pSWU30EE-2-11. These plasmids were transformed into the DK1622::pBJ-11 strain, which was used in the above construction process for the 11-2 spacer, forming series mutants. However, using the same cultivation and induction processes as mentioned above, we got no correct deletion strain with these three plasmids in screenings of approximately 200 colonies for each.

It is known that loading sgRNA into the binding complex of Cas9 and double-strand DNA substrates containing PAM-region is a crucial step in converting Cas9 into an active conformation capable of executing its nuclease function [6]. Ren et al. reported that the concentration level of Cas9, within a tested wide range, was not a critical factor for mutagenesis efficiency, while the amount of introduced sgRNA had a more profound impact on the in vivo mutagenesis and cell growth in eukaryotic *Drosophila* [46]. To increase the concentration of sgRNA in cells, we used the Golden Gate assembly [47] to replace the sgRNA promoter P_T7A1_ (58-bp in length) with a more powerful promoter P_pilA_ (167-bp in length) to produce the pUC-PXX series (Additional file 2: Figure S2), which were cloned into pSWU30E to form the pSWU30EE-3 plasmids containing the 11-1 to 11-4 spacers, respectively (Fig. 3a; Table 1). Similarly, we got no deletion mutant from the screening of approximately 100 colonies in each of the 11-1, -3 and -4 transformants. The result suggested that high levels of sgRNA were not critical to induce the production of DSB and recombination. Thus, within the tested range, the quantity of sgRNA was not a key factor influencing the sgRNA cleavage activity in the one-target CRISPR/cas9 experiment.

We further speculated that the quantity of Cas9 protein was probably an affecting factor for the genome edition in this high GC-content bacterium. As the DK1622 mutants integrated with the *cas9* gene could not grow beyond the 50 μM copper concentration (Fig. 1), we attempted to cultivate the mutants at a high copper concentration within one generation time, namely, approximately 4 h of incubation. The protocol was suggested to be able to obtain temporal high expressions of the Cas9 proteins, but reduce cell damage in long time incubation with the high expressions. After the short-time induction, the cultures were screened for the kan^−^ mutants on CTT agar plates using the kanamycin resistance. Interestingly, the transient high expressions of *cas9* at the 200 μM copper concentration led to the deletion efficiency of the myxovirescin gene cluster by sgRNA-11-2 approaching to 15.3 ± 3% of the bacterial population (Fig. 5b). At the concentration of 200 μM copper, the deletion rates by sgRNA11-3, -4 also reached to 0.67 and 2.33%, respectively. However, in the case of sgRNA11-1, there was still no positive colony from the screening of 200 colonies after the transient induction at the concentration of 200 μM copper ion.

### Optimization of 20 nt sgRNA spacers for the deletion of the myxalamid gene cluster

The above results not only indicated that temporally highly expressed *cas9* gene promoted the deletion efficiency, but also that the sequence features of a 20-nt spacer markedly affected the deletion rate of large fragments. This phenomenon also occurred in other bacteria, for example, in the assays of sgRNA contributions to cleavage efficiency in the deletion of a gene in *S. coelicolor*, where the highest deletion rate (60%) of one spacer was approximately two times higher than that of the other two spacers [19]. In their studies, Huang et al. designed the three 20 nt spacers inside the 1-kb ORF. However, the four spacers from 11-1 to 11-4 were designed flanking the target biosynthetic gene cluster, with greatly varied distances to the left and right homologous arms (referred to Fig. 5a), which probably caused the markedly different cleavage efficiencies of these sgRNA. In addition, we compared parameters of the four spacers in the sgRNA sequences (sgRNA11-1 to sgRNA11-4) (Table 1). While the GC content of the 11-1 spacer sequence was 50%, the other three were 75%. All of these four spacers had no hairpin structure. Interestingly, the free energy changes of the four sgRNA spacers were in the same order of the cleavage efficiency, i.e. 11-2 > 11-4 > 11-3 > 11-1. The free energy changes were small between 11-2, 11-3 and 11-4 spacers, but their cleavage efficiency differences were significant (Fig. 5b).

Lots of 20 nt spacers have been evaluated of the nucleotide preference at each position in the sequence across bacteria to human cell lines. If P values of the non-preferred nucleotides were set to 0.05, we could summarize a degenerate optimal spacer sequence 5′NVH-N2-BNH-N2-H-N5-HHNB-TGG (H: A/T/C, V: G/A/C, B: G/T/C, N: any nucleotide; the C-terminus TGG is the PAM motif) from findings by Liu et al. in mouse cell line [24]. However, the degenerate spacer sequence was not always consistent with those reported efficient spacer sequences, for example, the results published by Doench et al. [53], from which an optimal 20 nt spacer sequence 5′-NND-N5-H-N4-HNHNVVD-VGG (D: G/A/T, the others are the same as the above) could be proposed by setting the P value of non-preferred nucleotides to <0.01. These two degenerate optimal spacer sequences were greatly different. In our four spacer sequences, the 11-2 and 11-4 spacers fitted the degenerate spacer from Liu et al. [24] best, and then 11-1, while the 11-3 spacer had the many disfavored nucleotides (Table 1). Notably, when analyzing the rationally designed spacer sequences [24], we found that the spacer sequences in the highly active sgRNAs also had high free energies. Accordingly, we suggested that, in addition to the features of nucleotide preference, which might be varied in different contexts, the free energy of a spacer sequence was a critical factor to affect the cleavage efficiency.

**Fig. 6:**
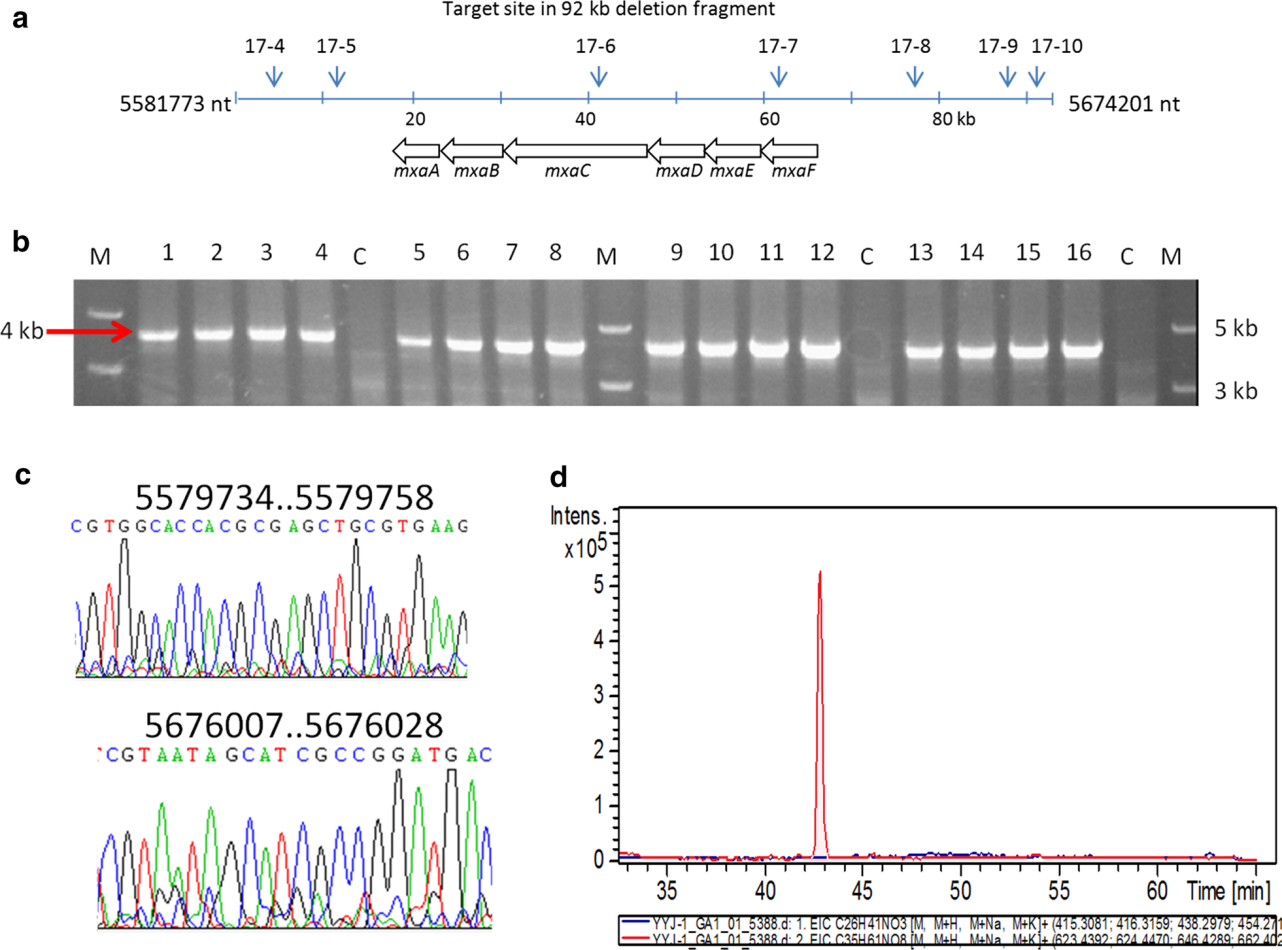
Large fragment deletion of the myxalamid biosynthetic gene cluster in *M. xanthus* DK1622 using the optimized spacer 17-6. **a** The seven sgRNA target sites (17-4 to 17-10) were revealed in or near the myxalamid gene cluster. The genes involved in the biosynthesis of myxalamid are shown in *arrows*. The positions of the guide sequence are indicated. **b** Identification of mutant strains by agarose gel electrophoresis of PCR product. The 4.0-kb positive fragment (shown with *red arrow*) appeared in different mutants (1–16), but not in DK1622 (**c**). **c** Chromatograms of the 4.0-kb PCR product DNA sequences, sequenced using the PCR primers as the sequencing primers. **d** EIC of myxalamid A ([*M*+H]^+^ *m/z* 416.31) from DK1622 (*red*) and mutant (*blue*)

We employed the proposed principle of 20 nt spacer for the deletion of the myxalamids biosynthetic gene cluster. We used PAM-containing 5′actgg to search 23-nt sequence that fits the 5′NVH-N2-BNH-N2-H-N5-HHACTGG architecture, and revealed seven qualified 20 nt spacers from one strand in the targeted 92 kb fragment of the myxalamids biosynthetic gene cluster (Fig. 6a; Table 1). All these seven spacers completely fitted the degenerate sequence. However, the free energy of these spacers was ranged from −35.8 to −49.3 kcals/mol, and the 17-6 spacer, which targeted the polyketide synthase gene *mxaC*, had the highest free energy. Similarly, the GC contents of these spacers were also varied, ranging from 55 to 80%. Two spacers (17-6 and 17-8) formed hairpins in the seed sequence but with different free energies, 49.3 and 39.3 kcals/mol. According to the above proposed principles, we selected the 17-6 spacer sequence to construct the pBJ-17 plasmid, which was further transformed into the wild type strain DK1622, producing strain DkpBJ17. Using the above protocol, we successfully got five kan^−^ strains from 35 colonies, and the deletion rate in these five mutants was confirmed to be 100% by PCR and sequencing (Fig. 6b, c). No myxalamid was produced in the mutants (Fig. 6d). The deletion efficiency of the 92-kb biosynthetic gene cluster for myxalamids thus reached to 14.3% of the bacterial population.

### Effects of the deletion of genes for secondary metabolites on the yields of others

In *M. xanthus* DK1622, there are 24 gene clusters for the biosynthesis of secondary metabolites, and six compounds have been identified and correlated to their gene clusters (Additional file 4: Figure S4). Most of these 24 gene clusters are of polyketide synthase (PKS) and/or nonribosomal peptide synthetase (NRPS), which are suggested to be interrelated because of complex regulation network, similar substrate units and transportation. To investigate effects of the deletions on the biosyntheses of other secondary metabolites, we compared the relative productions of some known secondary metabolites, i.e. myxalamids, myxovirescin (TA), myxoprincomide and DKxanthenes [48] in *M. xanthus* DK1622 and the deletion mutants of myxalamid or myxovirescin genes based on the LC–MS semiquantitative analysis. To avoid the influence from cultivation, we selected the yield of DKxanthene 534 as the standard to estimate ratio changes of the metabolites in the same strain.

**Fig. 7:**
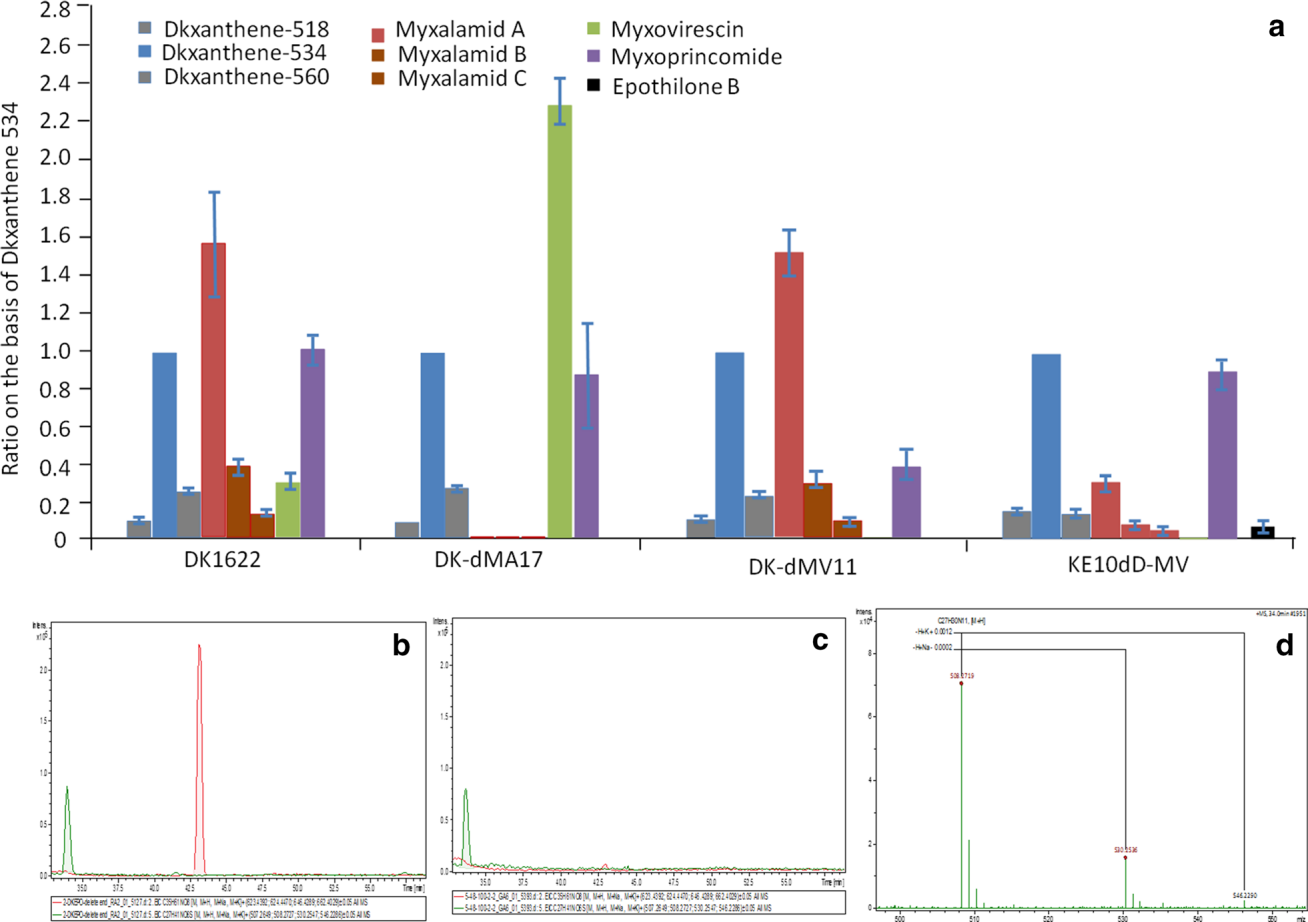
Comparison of the yields of secondary metabolites in *M. xanthus* DK1622 and epothilone-producing mutant with the deletion of myxalamid or myxovirescin genes. **a** In each single strain, the yield of DKxanthene 534 was set as standard, and the ratios of the yields of other metabolites were provided. Experiments were performed in triplicate. *Error bars* indicate standard deviations. DK-dMA17, the myxalamid-deleted mutant from DK1622; DK-dMV11, the myxovirescin-deleted mutant from DK1622; KE10dD-MV, the myxovirescin-deleted mutant from the epothilone-producing strain KE10. **b** EIC of myxovirescin A ([*M*+H]^+^ *m/z* 624.44, *red*) and epothilone B ([*M*+H]^+^ *m/z* 508.27, *green*) in KE10Dp11t. **c** EIC of myxovirescin A ([*M*+H]^+^ *m/z* 624.44, *red*) and epothilone B ([*M*+H]^+^ *m/z* 508.27, *green*) from KE10dD-MV. **d** The fragmentation patterns of epothilone B produced in the KE10Dp11t and KE10dD-MV with the correct molecular weight minus the H^+^, Na^+^, K^+^ (epothilone B = 507)

Among the analyzed compounds, myxalamid A had the largest yield in DK1622, reaching 1.5 times of DKxanthene 534, while myxoprincomide had a similar yield as DKxanthene 534, but myxovirescin gave the lowest yield, approximately one-third of DKxanthene 534 (Fig. 7a). The productions of DKxanthene 534 and myxalamid A homologues were greatly higher than their homologues, which were similar to the production of myxovirescin. In the myxalamid-deleted mutant (DK-dMA17), the yield ratio between myxoprincomide and DKxanthene 534 was almost unchanged, but the ratio between myxovirescin and DKxanthene 534 greatly increased to approximately 6.4-folds of the ratio in the wild type strain (Fig. 7a). After the deletion of myxovirescin, the yield ratio between DKxanthene 534 and myxalamid A was almost unchanged in the mutant DK-dMV11, but the yield of myxoprincomide became approximately one-third of DKxanthene 534. Notably, the ratios between the homologues of myxalamid or DKxanthene were almost unchanged in the mutants.

In our previous studies, we integrated the 56-kb gene cluster for the biosynthesis of antitumor polyketides epothilones into *M. xanthus* genome by transposition techniques [34]. To study effects of the deletion of endogenous biosynthetic gene cluster for secondary metabolites on the production of epothilones, we employed an epothilone-producing *M. xanthus* strain KE10 (Additional file 4: Figure S4) for the deletion of the myxovirescin gene cluster using a similar process based on pSWU30EE-2. Before the deletion, the resistance genes that were introduced into the genome accompanying with the epothilone gene cluster were removed from the epothilone-producing strain KE10 to obtain strain KE10D (Additional file 5: Figure S5). The pBJ-11 plasmid was further integrated into the genome of KE10D by the long right arm (1953 bp) to give the strain KE10Dp11. Then, the cas9/sgRNA-11 expression plasmid pSWU30EE-2-112 was integrated into the genome, producing the strain KE10Dp11t. The KE10Dp11t strain was still able to yield the major epothilone B analogue (Fig. 7b), and the yield of epothilone B was determined using HPLC–MS (Fig. 7c). After the induction by copper ion and the selection of kanamycin for KE10Dp11t using the above optimized method, we got the myxovirescin-deleted epothilone-producing strain KE10dD-MV (Fig. 7d) at about 8% of the bacterial population, a slightly less efficiency than that obtained in wild type DK1622. Like in the wild type strain DK1622, the deletion rate was 100% in the epothilone-producing *M. xanthus* strain. The correctness of kanamycin-negative colonies was confirmed by PCR and sequencing. From the ratios shown in Fig. 7a, the yield of epothilone B only occupied a small part of the repertoire of the secondary metabolites producible in the epothilone-producing *M. xanthus* cells. Notably, the relative yields of metabolites compared with DKxanthene 534 were mostly lower in KE10dD-MV than that in DK1622. However, compared with that in DK-dMV11, the myxovirescin-deletion epothilone-producer had lower yields of myxalamids, but higher yield of myxoprincomide (Fig. 7a). The above results showed that the deletion of biosynthetic gene clusters for secondary metabolites was able to significantly change the production of some other secondary metabolites in *M. xanthus*.

## Discussion

The researches on myxobacterial natural products gain great attentions [40, 49–51], but are normally limited by long generation time and laborious performances in cultivation and genetics. Compared with other ‘microbial factories’ like *S. coelicolor* or *E. coli*, the genetic manipulation methods available in *M. xanthus* are limited and normally laborious. In this study, we established an efficient CRISPR/Cas9 system for the deletion of large genome fragments in *M. xanthus* DK1622 after resolving some critical limits. For example, we employed pBJ113 to delete the 8.4-kb resistance-related genes that were introduced into the genome accompanying with the epothilone gene cluster (Additional file 5: Figure S5). After the kanamycin and galactose selections, we obtained strain KE10D with a deletion efficiency of 3.7%, which required lots of PCR to identify the recovery mutant. Comparatively, the recovered kanamycin resistant mutants by the proposed CRISPR/Cas9 deletion protocol were all the deletion mutants. In this protocol, we developed a strategy for temporally high expressions of Cas9 to decrease cytotoxicity of the endonuclease in *M. xanthus*, and optimized the deletion protocol by using a tRNA–sgRNA–tRNA strategy to ensure the correct targeting by sgRNA sequence. We found that the free energy of a 20 nt spacer sequence was positively correlated with the Cas9 cleavage efficiency. Because Cas9 has no energy-dependent helicase activity, the mechanism of the local DNA unwinding is still unknown, but probably relies upon thermally available energy [52]. We speculated that higher free energy of 20 nt spacer might help the formation of the RNA:DNA heteroduplex. Cas9 is not converted into a more active conformation for executing its nuclease function, but instead, the generated RNA:DNA heteroduplex might provide substrates for a more efficient activity nuclease activity of Cas9.

In theory, any specific 20 nt sequence followed by a PAM can be used to target the DNA sequence for the Cas9-induced directed cleavage, but the cleavage efficiencies of different spacers may be varied. There are three sequence features affecting the efficiency of 20 nt spacer, i.e. the GC content, secondary structure and nucleotide preference. Analysis of these sequence features, however, produced opposite conclusions. For example, the GC content within the range of 40–60% was proposed to be favored based on investigations in eukaryotic cells for a high gRNA activity [22, 24, 25, 46, 53, 54]. However, the studies in zebrafish embryos observed a positive correlation between GC content and indel frequency that was produced by the Cas9 cleavage via nonhomologous end joining repair system [23]. Thyme et al. comprehensively analyzed effects of intra-sgRNA interactions on cleavage efficiency, and proposed the presence of a hairpin structure inhibited cleavage [10]. In contrast, Liu et al. proposed that if the seed sequence is more likely to form secondary structure, the sgRNA has a higher chance of cleaving the target sequence [24]. The 8–12 PAM-proximal bases, namely the seed sequence of sgRNA, were proposed to be more important than the other spacer bases in the determination of targeting specificity [13, 22, 46, 53, 54]. However, from the evaluation of the cleavage activities of 218 sgRNAs using in vitro Surveyor assays in mouse cell line, Takummi and collaborators found that the nucleotides at either PAM-distal or PAM-proximal regions of spacer were significantly correlated with the on-target efficiency [24]. This is in line with the observation found in zebrafish that the PAM-distal sequences were involved in targeting cleavage and that the Cas9-target binding depended on the PAM-proximal sequences [55]. Liu et al. reported that the nucleotides at positions 2(T), 3(G), 6(A), 8(G), 11(G), 17(G), 18(G) and 20(A) were disfavored for cleavage activity based on their in vitro mismatch nuclease assay in mouse cells [24]. However, by investigating nucleotide preference at each position of the 20 nt spacer sequence and PAM in 1841 sgRNAs, Doench et al. found that the nucleotides at positions 3(C), 9(G), 14(G), 16(G), 18(T), 19(T) and 20(C) were disfavored [50]. The activity measurement of 1280 sgRNAs targeting 128 different genes in the zebrafish genome showed that the guanine enrichment and the adenine depletion increased sgRNA stability and activity [25], which were different from the above results of the disfavored nucleotides in 20 nt spacer sequences. Similarly, Gagnon et al. observed that G is favored, and A is disfavored in the 20th position [23]. Accordingly, from references, as well as from our results, different 20 nt spacer sequences significantly affect the Cas9 cleavage efficiency, probably depending on the editing genomes or experimental conditions. Herein, the free energy of spacer sequence plays a critical role.

In this study, we proposed a degenerate sequence for the design of an optimal 20 nt spacer from the evaluation of 20 nt spacer sequences and their cleavage efficiencies in references related to *Streptomyces* [19], mouse cell line [24] and human cell lines [7]. Our experimental results showed that the spacer formula was consistent with the finding of position-dependent nucleotide features of 20 nt spacer published by Huang et al. [16, 17], and was similar to the results obtained from the in vitro results of mouse cell line, if *P* values of non-preferred nucleotides were set to <0.05 [24]. This optimal degenerate sequence fits many efficient Cas9-induced DNA cleavage from bacteria to human. We suggested that the findings and proposals described in this paper were workable in different organisms, at least the Gram negative bacteria with high GC content.

## Methods

### Strains, media and reagents

Strains and plasmids are listed in Additional file 6: Table S1. *Escherichia coli* strain Top 10, used for plasmid cloning and maintenance, was grown in LB medium supplemented with tetracycline (5 µg/mL) and kanamycin (20 µg/mL) when needed. *Myxococcus xanthus* DK1622 and its mutant strains were grown in CTT medium with kanamycin (40 µg/mL) and/or Tetracycline (15 µg/mL) if needed.

The oligonucleotide primers used in this study (Additional file 7: Table S2) were synthesized by Sangon (Shanghai, China). KOD plus Neo DNA polymerase (Toyobo, Osaka, Japan) was used for high fidelity PCR amplification. Restriction digestion enzymes and ligase were purchased from Thermo Fisher Scientific (USA)and Takara (Dalian, China).

### Construction of plasmids

We designed a tRNA–sgRNA–tRNA chimeric gene for correct production of sgRNA using the previously described method [12] with modifications. This chimeric gene was under the control of the T7A1 constitutive promoter (Accession no. A15404) or *pilA* promoter (the complement sequence 7159461..7159631 in *M. xanthus* DK1622), along with the widely-used T7 terminator (Additional file 1: Figure S1). There are five Gly-tRNA genes in DK1622, and we selected the *MXAN_3066* gene (corresponding to the GGA codon) to integrate into the cassette. The tRNA–sgRNA–tRNA expression cassette following the promoter T7A1 was synthesized by Sangon Company (Shanghai, China) and cloned into the pUC57 vector to give the plasmid pUC57-TXX (Additional file 2: Figure S2). Golden Gate assembly was employed to substitute the T7A1 promoter to the *pilA* promoter, which was obtained by PCR amplification from the DK1622 genome, to produce the plasmid pUC-PXX (Additional file 2: Figure S2). This small circular pUC57-sgRNA (pUC-TXX or pUC-PXX) was used as a template for PCR amplification with the primer pair gScaff-F/gRNAX-R containing a 20-nt target spacer by KOD-Plus-Neo high fidelity DNA polymerase (Fig. 3b). After phosphorylation and self-ligation circularization of PCR fragment, we obtained plasmids with the tRNA–sgRNA–tRNA cassette targeting different sites. Then we cloned these cassettes into pSWU30EE series plasmids by double digestion with *Eco*RI and *Hin*dIII (Additional file 3: Figure S3).

The codon-optimized *cas9* gene was obtained by double digestion of plasmid pKCcas9d 6424 [19] with *Nde*I and *Eco*RI, and then ligated into pET28a digested with *Nde*I and *Eco*RI to yield pET28a–cas9 (Additional file 3: Figure S3). The constitutive P_pilA174_ promoter (the complementary sequence 7159458..7159631 in DK1622 genome; we added an additional ATG before the *cas9* gene) and the copper-inducible P_cuoA_ promoter (the complementary sequence 3976002..3976945 in DK1622 genome) were obtained from *M. xanthus* DK1622 using the primers PcuF4/PcuR4 and PilAF/PilAR, respectively. These two fragments were digested with *Xba*I and *Nde*I, and then ligated into pET28a–cas9 to produce plasmids pET28a–P_pilA174_-cas9 and pET28a–P_cuoA_-cas9, respectively. Finally, the vector pSWU30 was changed to pSWU30E at the *Eco*RI site, producing the pSWU30EE-1,-2,-3 plasmids.

To construct plasmids with homologous arms, pBJ113 was used by conventional cloning method with three restriction enzymes of *Eco*RI, *Xba*I and *Hin*dIII. To increase the homologous recombination efficiency, the length of homologous arms was designed to be 1.5–2.0 kb. To construct the plasmid pBJ11, the primer pair of S11U-1483F and S11U-2619R was used to amplify the left homologous arm, which was then digested by *Eco*RI + *Xba*I and ligated to vector pBJ113 to give plasmid pBJ113-11U. The primer pair of S11D-141F and S11D-2093R was used to amplify the right homologous arm, which was digested by *Xba*I + *Hin*dIII and ligated to pBJ113-11U to give plasmid pBJ11. By the same method, we constructed plasmid pBJ17.

The above plasmid constructions were confirmed by restriction analysis and sequencing.

### Electro-transformation of *M. xanthus*

Homologous recombination plasmid pBJ113 series and *cas9* expression plasmid pSWU30EE series were stepwise electro-transformed into *M. xanthus* cells. The constructed plasmids were introduced into *M. xanthus* cells using electroporation as described previously [56] with small modifications. We firstly integrated the pBJ113 series plasmids into DK1622 genome by homologous recombination. The pBJ113 series plasmids containing two homologous arms located at the both ends of a selected large gene cluster were electro-incorporated into DK1622, and then resistant mutants were screened on CTT+kan agar plate. After confirmation of integration type (left or right homologous arm) by PCR amplification, we then integrated the pSWU30EE series plasmids into genome by site-specific recombination. The P_pilA_-driven pSWU30EE-1 or P_cuoA_-driven pSWU30EE-2, -3 plasmids were transferred into kanamycin-positive mutant strains. The transformants were screened on CTT+Kan+Tet agar plate. After 4–5 days of incubation at 30 °C, we transferred the resistant strains into fresh CTT liquid medium.

### Induction of *cas9* expression by copper ion

Double integrated mutants were grown on agar plate containing CTT+Kan+Tet medium without copper for 3–4 days. Then the cells were transferred into a fresh CTT liquid medium supplemented with 25 μM concentration of copper, and adjusted to an optical density of 0.05 at 600 nm (OD_600_). After 48 h of incubation at 30 °C (before reaching the end of logarithmic phase), the cells were harvested and mixed in soft agar, spread on CTT agar plates to screen the survival colonies.

Induction of temporally high expression was performed as below. The mutants were cultivated in CTT+Kan+Tet medium without copper for 48 h at 30 °C. The cells were harvested by centrifugation, washed one time with fresh liquid CTT medium, and then inoculated into fresh liquid CTT medium supplemented with the copper concentrations needed. After approximately 4 h of incubation (one generation time) at 30 °C, the cells were mixed in soft agar for plating on CTT agar plates.

### Screening of the deletion mutants by kanamycin selection and PCR sequencing

After induction by copper ion, the cells were spread on CTT agar plates, and incubated for 5–7 days. The single colonies were transferred simultaneously to new CTT agar plates supplemented with and without kanamycin. Normally, 100 or 200 colonies were transferred for the screening. The percentage of the colonies that were unable to grow on CTT+kan agar plate in the total colonies on CTT plates were calculated as the cleavage efficiency. The kan^−^ colonies were further determined by PCR amplification and sequencing for the deletion of the genome fragments.

### Preparation of *M. xanthus* sample sets for the assay of secondary metabolites

*Myxococcus xanthus* strains were cultivated in 50-mL CTT medium supplemented with 2% XAD-16 resin (Sigma-Aldrich). After 96 h of shaking at 200 rpm and 30 °C, the cells and XAD resin were harvested by centrifugation (20 min, 5000 rpm, 20 °C). The pellet was extracted with 3 mL methanol overnight, and the extracts were dried in vacuum and resuspended in 3 mL distilled methanol. Three replicates were performed for each experiment.

### Identification of compounds using high-resolution HPLC–MS/MS system

HPLC–MS/MS was performed on a rapid separation liquid chromatography system (Dionex, UltiMate3000, UHPLC) coupled with an ESI-Q-TOF mass spectrometer (Bruker Daltonics, Impact HD). Separation of compounds was performed on an YMC-Packpro C18 column (250 mm × 4.6 mm I.D., particle size 5 µm) with mobile phase system of 0.1% formic acid (Sigma) in Mili-Q filtered water (A) and 0.1% formic acid (Sigma) in acetonitrile (Fisher Scientific) (B). The following gradient program was applied at a flow rate of 0.75 mL/min: 0–5 min 95% A + 5% B; 5–45 min 95–5% A + 5–95% B; 45–60 min 5% A + 95% B.

The MS/MS analysis was performed by otofControl software (Bruker Daltonics) under the following conditions: ESI-positive mode for scanning, enhanced quadratic mode for calibration, nebulizer gas nitrogen with 1 bar, dry gas nitrogen with 8 L/min, probe temperature of 180 °C, full scan mass range from 50 to 1500 *m/z*, auto MS/MS for each precursor ion. The individual extraction chromatograms and their abundances in area were confirmed by the formulas, *m/z* values and MS/MS fragments information using DataAnalysis software (Bruker Daltonics). The production levels of compounds were averaged from three independent cultivations and extractions.

## Additional files

**Additional file 1: Figure S1.** The sequence of artificial tRNA–sgRNA–tRNA transcription cassette (373 bp).

**Additional file 2: Figure S2.** The plasmid construction of model cassette pUC57–sgRNA. Golden Gate Assembly was employed to replace the T7A1 promoter of the sgRNA cassette (in the pUC-TXX plasmids) with the pilA promoter (in the pUC-PXX plasmids).

**Additional file 3: Figure S3.** Scheme and workflow for the construction of strains DK-Cas9 and DK-Cas9–sgRNA and the initial cas9 expression plasmid pSWU30EE-2.

**Additional file 4: Figure S4.** Genomic map of *M. xanthus* KE10, indicating the numbers and types, as well as the locations of secondary metabolite gene clusters on genome. The gene clusters that could be correlated to the produced secondary metabolites are indicated, the two gene clusters to be deleted in this study are shown in yellow, and the four other known secondary metabolite gene clusters shown in red. The inserted epothilone biosynthetic gene cluster shown in green at position 0.09 Mb.

**Additional file 5: Figure S5.** Deletion of the vector and unrelated resistant genes near epothilone gene cluster in epothilone-producing strain KE10. A. A 2.0-kb homologous arm locating in the right end of epothilone gene cluster (orf A shown in green) and a 1.5 kb homologous arm locating in the right end from DK1622 (part of MXAN_0084 and MXAN_0085 shown in gray) were amplified and cloned into suicide vector pBJ113 for the deletion. B. Identification of mutant strains by PCR amplification using the primers 11, 16 (primer locations are shown in A panel). The 3.0-kb band in lanes 7 and 10 was the positive PCR product. C. Identification of mutant strains by PCR amplification using the primers 15, 16 (primer locations are shown in A panel). The 0.9-kb band disappeared in lanes 7 and 10.

**Additional file 6: Table S1.** Strains and plasmids used in this study.

**Additional file 7: Table S2.** Primers used in this study.
