# Supplementary figures and images for: Increasing on-target cleavage efficiency for CRISPR/Cas9-induced large fragment deletion in Myxococcus xanthus
Source: Microb Cell Fact. 2017 Aug 16;16:142. doi: 10.1186/s12934-017-0758-x (PMC5559782; doi:10.1186/s12934-017-0758-x)

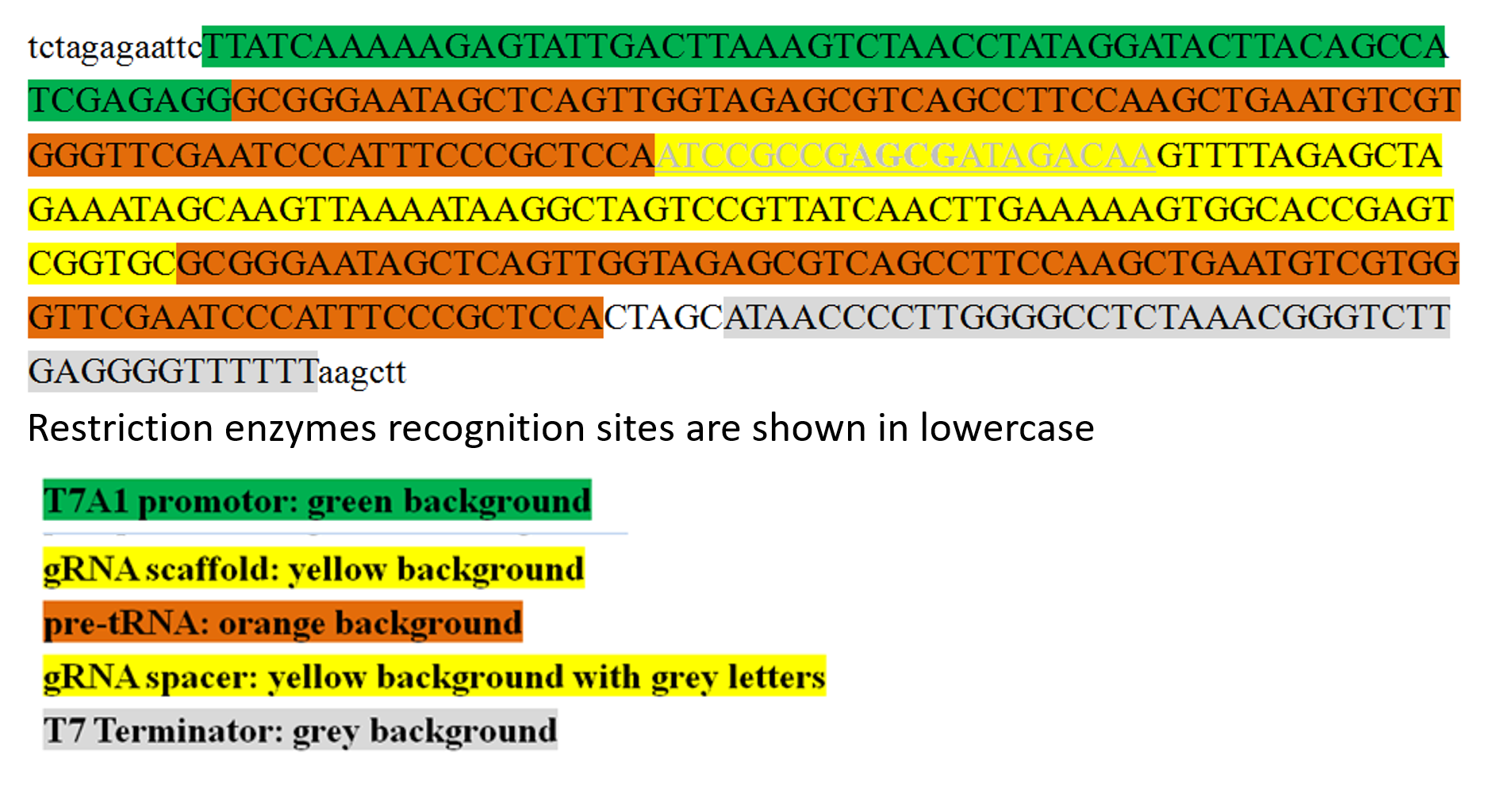

Supplement: Supplementary file 1 — Additional file 1: Figure S1. The sequence of artificial tRNA–sgRNA–tRNA transcription cassette (373 bp). [file 12934_2017_758_MOESM1_ESM.tif]

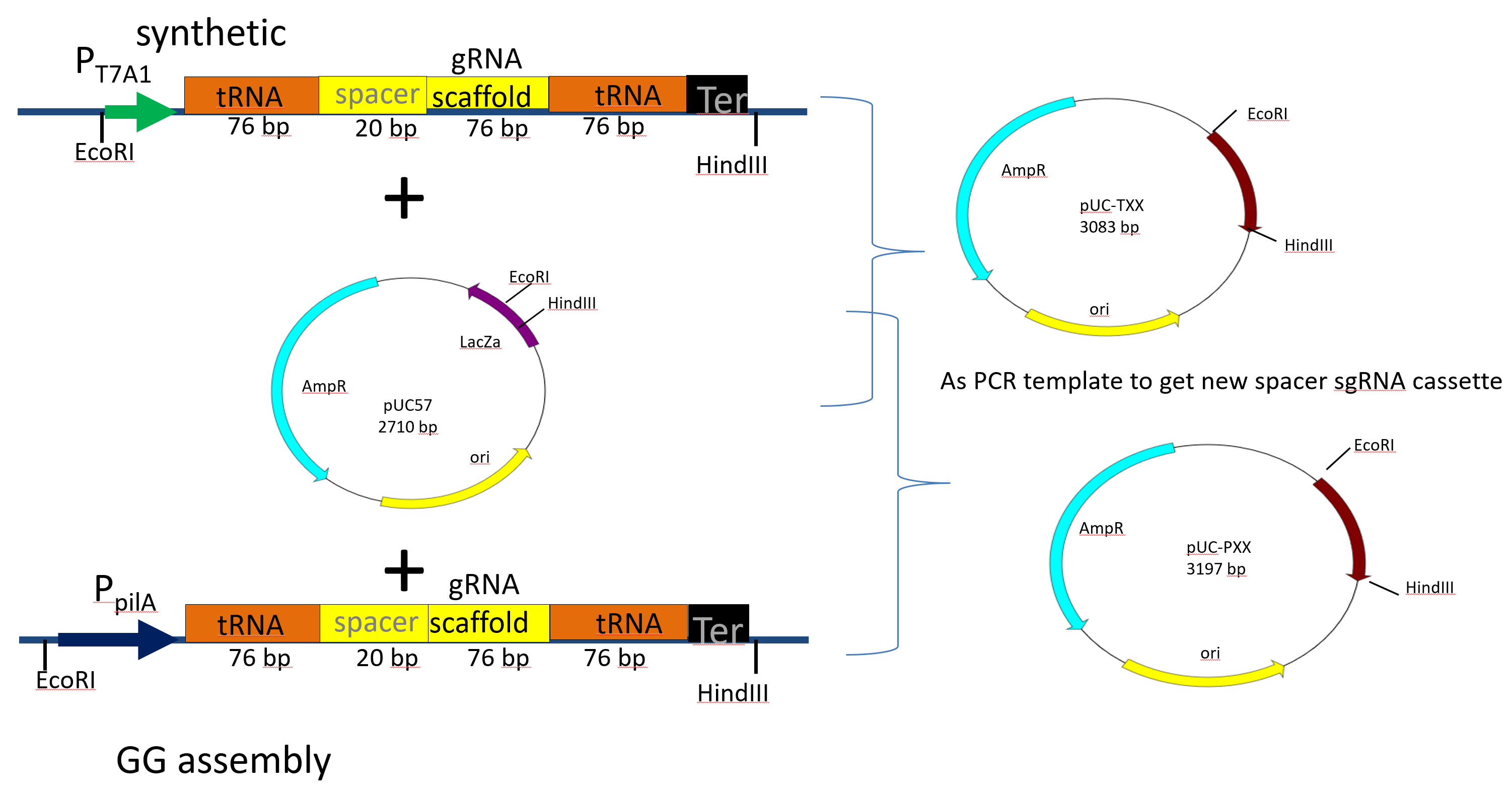

Supplement: Supplementary file 2 — Additional file 2: Figure S2. The plasmid construction of model cassette pUC57–sgRNA. Golden Gate Assembly was employed to replace the T7A1 promoter of the sgRNA cassette (in the pUC-TXX plasmids) with the pilA promoter (in the pUC-PXX plasmids). [file 12934_2017_758_MOESM2_ESM.tif]

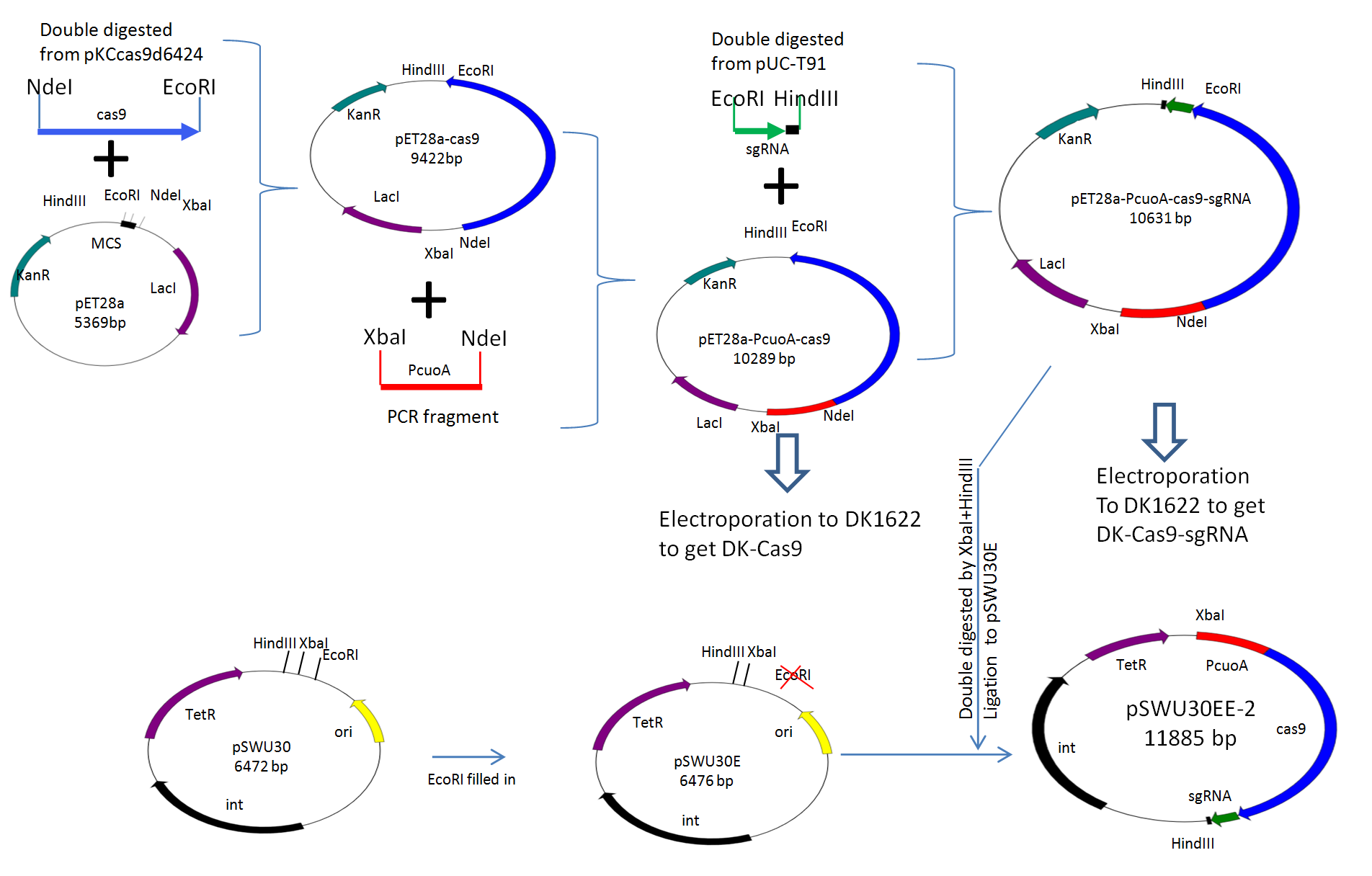

Supplement: Supplementary file 3 — Additional file 3: Figure S3. Scheme and workflow for the construction of strains DK-Cas9 and DK-Cas9–sgRNA and the initial cas9 expression plasmid pSWU30EE-2. [file 12934_2017_758_MOESM3_ESM.tif]

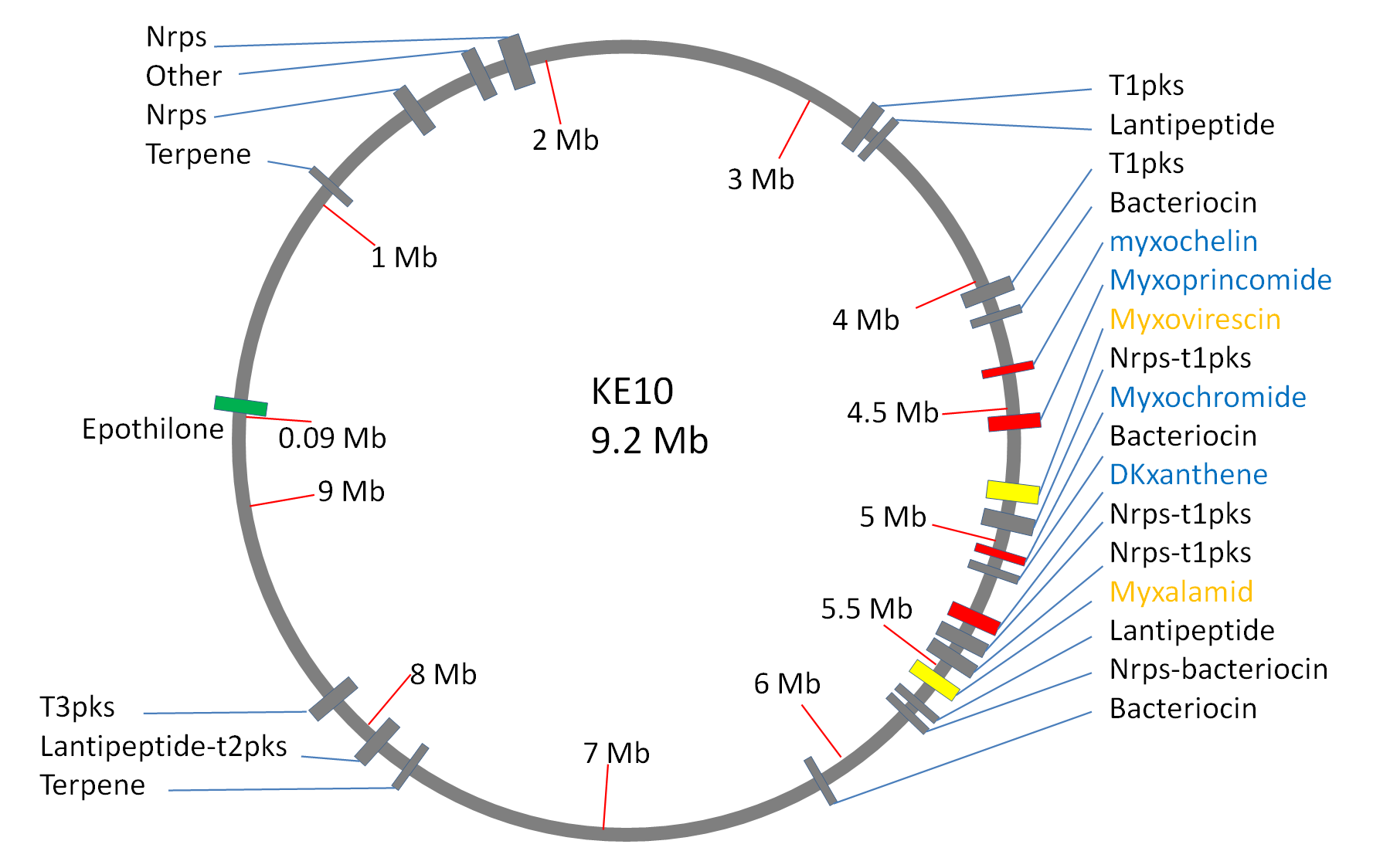

Supplement: Supplementary file 4 — Additional file 4: Figure S4. Genomic map of M. xanthus KE10, indicating the numbers and types, as well as the locations of secondary metabolite gene clusters on genome. The gene clusters that could be correlated to the produced secondary metabolites are indicated, the two gene clusters to be deleted in this study are shown in yellow, and the four other known secondary metabolite gene clusters shown in red. The inserted epothilone biosynthetic gene cluster shown in green at position 0.09 Mb. [file 12934_2017_758_MOESM4_ESM.tif]

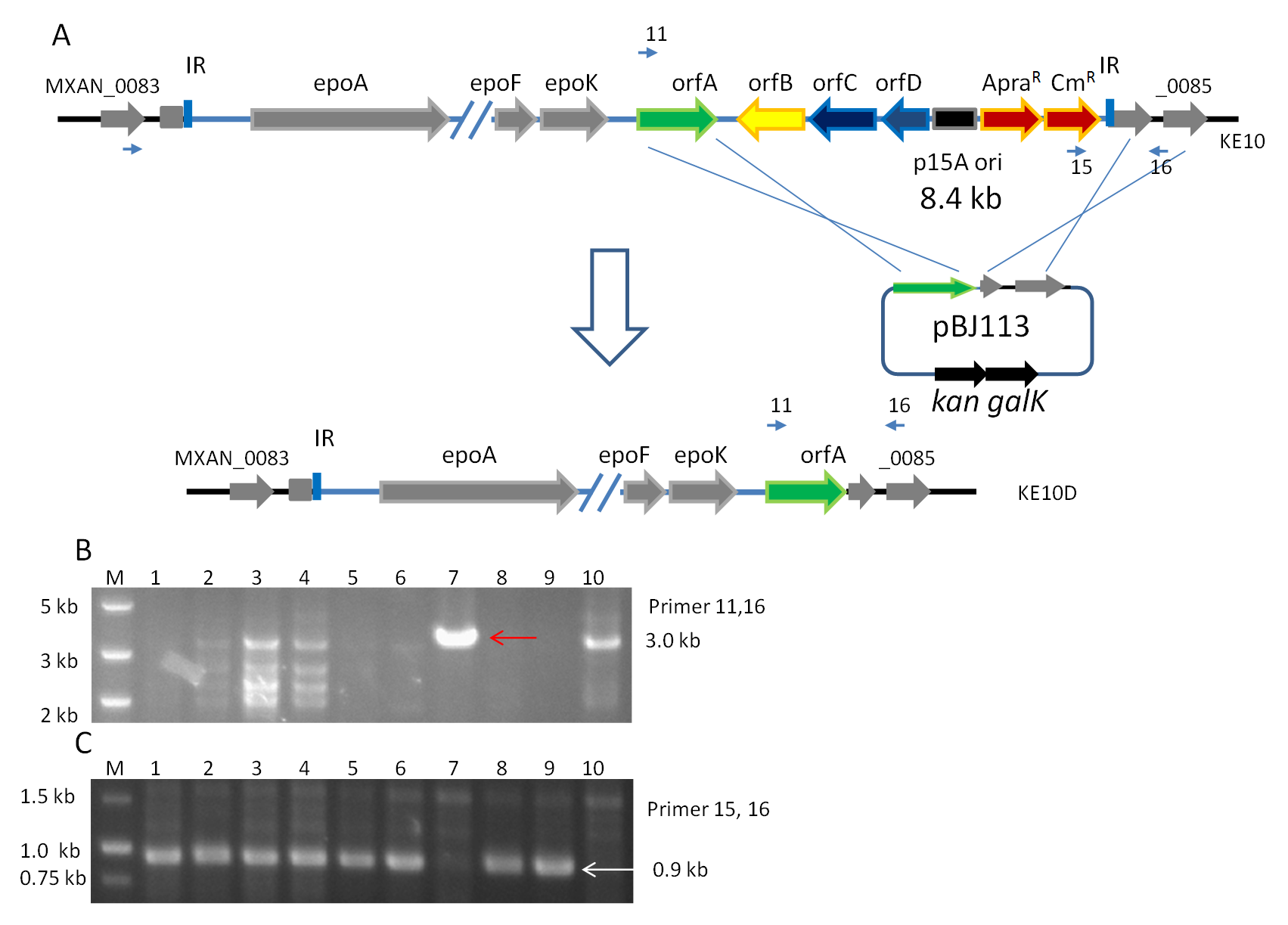

Supplement: Supplementary file 5 — Additional file 5: Figure S5. Deletion of the vector and unrelated resistant genes near epothilone gene cluster in epothilone-producing strain KE10. A. A 2.0-kb homologous arm locating in the right end of epothilone gene cluster (orf A shown in green) and a 1.5 kb homologous arm locating in the right end from DK1622 (part of MXAN_0084 and MXAN_0085 shown in gray) were amplified and cloned into suicide vector pBJ113 for the deletion. B. Identification of mutant strains by PCR amplification using the primers 11, 16 (primer locations are shown in A panel). The 3.0-kb band in lanes 7 and 10 was the positive PCR product. C. Identification of mutant strains by PCR amplification using the primers 15, 16 (primer locations are shown in A panel). The 0.9-kb band disappeared in lanes 7 and 10. [file 12934_2017_758_MOESM5_ESM.tif]
