# Supplementary material for: Increasing on-target cleavage efficiency for CRISPR/Cas9-induced large fragment deletion in Myxococcus xanthus
Source: Microb Cell Fact. 2017 Aug 16;16:142. doi: 10.1186/s12934-017-0758-x (PMC5559782; doi:10.1186/s12934-017-0758-x)
Supplement: Supplementary file 6 — Additional file 6: Table S1. Strains and plasmids used in this study. [file 12934_2017_758_MOESM6_ESM.docx]

Table S1 Strains and plasmids used in this study

| Strain and plasmid | characteristics | references or Sources |
| --- | --- | --- |
| Strain |  |  |
| E. coli |  |  |
| Top 10 | F– mcrA Δ(mrr-hsdRMS-mcrBC) Φ80lacZΔM15 ΔlacX74 recA1 araD139 Δ(ara leu) 7697 galU galK rpsL (StrR) endA1 nupG | Life technologies |
| M. xanthus |  |  |
| DK1622 | Wild type | Kaiser, 1979 |
| KE10 | DK1622 MXAN_0084∷epo. Heterologously epothilone-producing strain, M.xanthus DK1622 as host, apr^R^, Cm^R^ | Zhu, et. al., 2015 |
| KE10D | KE10Δ(est-aprR-cmR).The flanking sequence containing esterase, apr^R^ and cm^R^ was deleted from KE10. | This study |
| KE10Dp11 | KE10-D∷pBJ113-S11UD, kan^R^ | This study |
| KE10Dp11t | KE10-D∷pBJ113-S11UD∷pSWU30E-P_cuoA_-cas9- P_T7A1_-sgRNA-11-2, kan^R^, Tet^R^. | This study |
| KE10dD-MV | KE10-DΔ(4711144-4820044 nt)∷pSWU30E-P_cuoA_-cas9-P_T7A1_-sgRNA-11-2, Tet^R^. | This study |
| DKcas9 | DK1622∷pET28a-P_cuoA_-cas9, integrated by 1kb copper promoter homologous arm, kan^R^ | This study |
| DKcas9-tsg91 | DK1622∷pET28a-P_cuoA_-Scocas9-P_T7A1_-sgRNA-9-1, integrated by 1kb copper promoter homologous arm, Kan^R^ | This study |
| DKpBJ9 | DK1622∷pBJ113-S9UD, integrated by 2kb homologous arms of myxochelin gene cluster, kan^R^ | This study |
| DKpBJ11 | DK1622∷pBJ113-S11UD, integrated by 2 kb homologous arms of myxovirescin gene cluster, kan^R^ | This study |
| DKpBJ17 | DK1622∷pBJ113-S17UD, integrated by 2kb homologous arms of myxalamid gene cluster, kan^R^ | This study |
| DKpBJ9-Ptsg92 | DK1622∷pBJ113-S9UD∷pSWU30E-P_PilA_-cas9-P_T7A1_- sgRNA-9, kan^R^, Tet^R^. | This study |
| DKpBJ11-Ptsg112 | DK1622∷pBJ113-S11UD∷pSWU30E-P_PilA_-cas9- P_T7A1_- sgRNA-11-2, kan^R^, Tet^R^. | This study |
| DKpBJ9-tsg92 | DK1622∷pBJ113-S9UD∷pSWU30E-P_cuoA_-cas9-P_T7A1_- sgRNA-9, kan^R^, Tet^R^. | This study |
| DKpBJ11-tsg112 | DK1622∷pBJ113-S11UD∷pSWU30E-P_cuoA_-cas9- P_T7A1_- sgRNA-11-2, kan^R^, Tet^R^. | This study |
| DKpBJ17-tsg17 | DK1622∷pBJ113-S17UD∷pSWU30E-P_cuoA_-cas9- P_T7A1_- sgRNA-17, kan^R^, Tet^R^. | This study |
| DKpBJ11-tsg111 | DK1622∷pBJ113-S11UD∷pSWU30E-P_cuoA_-cas9- P_T7A1_- sgRNA-11-1, kan^R^, Tet^R^. | This study |
| DKpBJ11-tsg113 | DK1622∷pBJ113-S11UD∷pSWU30E-P_cuoA_-cas9- P_T7A1_- sgRNA-11-3, kan^R^, Tet^R^. | This study |
| DKpBJ11-tsg114 | DK1622∷pBJ113-S11UD∷pSWU30E-P_cuoA_-cas9- P_T7A1_- sgRNA-11-4, kan^R^, Tet^R^. | This study |
| DKpBJ11-psg111 | DK1622∷pBJ113-S11UD∷pSWU30E-P_cuoA_-cas9- P_pilA_- sgRNA-11-1, kan^R^, Tet^R^. | This study |
| DKpBJ11-psg112 | DK1622∷pBJ113-S11UD∷pSWU30E-P_cuoA_-cas9- P_pilA_- sgRNA-11-2, kan^R^, Tet^R^. | This study |
| DKpBJ11-psg113 | DK1622∷pBJ113-S11UD∷pSWU30E-P_cuoA_-cas9- P_pilA_- sgRNA-11-3, kan^R^, Tet^R^. | This study |
| DKpBJ11-psg114 | DK1622∷pBJ113-S11UD∷pSWU30E-P_cuoA_-cas9- P_pilA_- sgRNA-11-4, kan^R^, Tet^R^. | This study |
| DK-dMV11 | DK1622 Δ(4711144-4820044 nt)∷pSWU30E-P_cuoA_-cas9- P_T7A1_-sgRNA-11, Tet^R^. | This study |
| DK-dMA17 | DK1622 Δ(5581774-5674200 nt)∷pSWU30E-P_cuoA_-cas9-P_T7A1_-sgRNA-17, Tet^R^. | This study |
| plasmid |  |  |
| pET28a | Cloning vector, Kan^R^ |  |
| pBJ113 | Gene replacement vector with KG cassette, Kan^R^ | Julien, et.al., 2000 |
| pSWU30 | Cas9 and sgRNA expression vector, Site specific integration vector with Mx8 attB, Tet^R^ | Wu, et. al.,1997 |
| pKCcas9d6424 | Containing codon-optimized cas9 gene between NdeI and EcoRI site, Apr^R^. | Huang, et. al., 2015 |
| pMAT3 | P_cuoA_ Mx8 *attP int* Tet^R^ , derived from pSWU30 | Gómez-Santos, et.al., 2012 |
| pSWU30E | EcoRI site of pSWU30 was filled in | This study |
| pECas9 | pET28a-cas9 | This study |
| pET-Cas9 | pET28a-P_cuoA_-cas9 | This study |
| pEPilCas9 | pET28a- P_pilA174_ -cas9 | This study |
| pET-Cas9-sgRNA | pET28a-P_cuoA_-cas9-P_T7A1_-sgRNA-9-1 | This study |
| pUC-T91 | Artificially synthesized tRNA-sgRNA cassette digested by EcoRI+HindIII, AmpR. pUC57 as a backbone | This study |
| pUC-T92 | tRNA-sgRNA cassette, driven by promoter T7A1 | This study |
| pUC-T111 | tRNA-sgRNA cassette, driven by promoter T7A1 | This study |
| pUC-T112 | tRNA-sgRNA cassette, driven by promoter T7A1 | This study |
| pUC-T113 | tRNA-sgRNA cassette, driven by promoter T7A1 | This study |
| pUC-T114 | tRNA-sgRNA cassette, driven by promoter T7A1 | This study |
| pUC-T176 | tRNA-sgRNA cassette, driven by promoter T7A1 | This study |
| pUC-P111 | tRNA-sgRNA cassette, driven by promoter pilA | This study |
| pUC-P112 | tRNA-sgRNA cassette, driven by promoter pilA | This study |
| pUC-P113 | tRNA-sgRNA cassette, driven by promoter pilA | This study |
| pUC-P114 | tRNA-sgRNA cassette, driven by promoter pilA | This study |
| pBJ-9 | pBJ113-S9UD, upstream and downstream of two homologous arms cloned flanking the myxochelin gene cluster | This study |
| pBJ-11 | pBJ113-S11UD, upstream and downstream of two homologous arms cloned flanking the TA gene cluster | This study |
| pBJ-17 | pBJ113-S17UD, upstream and downstream of two homologous arms cloned flanking the myxalamid gene cluster | This study |
| pSWU30EE-1-91 | pSWU30-P_pilA_-cas9- P_T7A1_-sg9-1 | This study |
| pSWU30EE-1-112 | pSWU30-P_pilA_-cas9- P_T7A1_-sg11-2 | This study |
| pSWU30EE-2-91 | pSWU30-P_cuoA_-cas9-P_T7A1_-sg9-1 | This study |
| pSWU30EE-2-111 | pSWU30-P_cuoA_-cas9-P_T7A1_-sg11-1 | This study |
| pSWU30EE-2-112 | pSWU30-P_cuoA_-cas9-P_T7A1_-sg11-2 | This study |
| pSWU30EE-2-113 | pSWU30-P_cuoA_-cas9-P_T7A1_-sg11-3 | This study |
| pSWU30EE-2-114 | pSWU30-P_cuoA_-cas9-P_T7A1_-sg11-4 | This study |
| pSWU30EE-3-111 | pSWU30E-P_cuoA_-cas9-P_pilA_-sg11-1 | This study |
| pSWU30EE-3-112 | pSWU30E-P_cuoA_-cas9-P_pilA_-sg11-2 | This study |
| pSWU30EE-3-113 | pSWU30E-P_cuoA_-cas9-P_pilA_-sg11-3 | This study |
| pSWU30EE-3-114 | pSWU30E-P_cuoA_-cas9-P_pilA_-sg11-4 | This study |

Julien, B., Kaiser, A. D., & Garza, A. (2000). Spatial control of cell differentiation in Myxococcus xanthus. *Proc Natl Acad Sci U S A, 97*(16), 9098-9103.

Kaiser, D. (1979). Social gliding is correlated with the presence of pili in Myxococcus xanthus. *Proc Natl Acad Sci U S A, 76*(11), 5952-5956.

Wu, S. S., Wu, J., & Kaiser, D. (1997). The Myxococcus xanthus pilT locus is required for social gliding motility although pili are still produced. *Mol Microbiol, 23*(1), 109-121.
