# Supplementary material for: Increasing on-target cleavage efficiency for CRISPR/Cas9-induced large fragment deletion in Myxococcus xanthus
Source: Microb Cell Fact. 2017 Aug 16;16:142. doi: 10.1186/s12934-017-0758-x (PMC5559782; doi:10.1186/s12934-017-0758-x)
Supplement: Supplementary file 7 — Additional file 7: Table S2. Primers used in this study. [file 12934_2017_758_MOESM7_ESM.docx]

Table S2 primers used in this study

| Name | Sequence (5’-3’) | Target/comment |
| --- | --- | --- |
| PcuF4 | gcTCTAGATTCAACGGCATTCATGGGGCTTCC | Copper promoter |
| PcuR4 | ggGAATTCCATATGGAAGCCTCTTCACGAATGGATG |  |
| PilAF2 | gcTCTAGATCGTCAGCGTCACTGCCGAATTTTGTC | pilA promoter |
| PilAR2 | GGGAATTCCATATGCATGGGGGTCCTCAGAGAAGGTTGC |  |
| ScoF | aaCATATGATGGACAAGAAGTACTCCATC | Cas9 |
| Sco endRT1 | cgGAATTCATAAAACGAAAGGCCCAGTCTTTCGACTGAGCCTTTCGTTTTATaaTCAGTCGCCGCCCAGCTGGGAGA | Cas9+terminator |
| Sco endRT2 | cgGAATTCAAAAAGGCCATCCGTCAGGATGGCCTTCTGTCAGTCGCCGCCCAGCTGGGAGA | Cas9+terminator |
|  |  |  |
| S9U-60F | cgGAATTCCCCTGACGCAGTAGTGGTATT | Left arm of myxochelin gene cluster |
| S9U-1299R | gcTCTAGAAGCCCTGTTGGGTGTTCC |  |
| S9D-2274f | gcTCTAGAGGGGTTGCGGGAGAAACAA | Right arm of myxochelin gene cluster |
| S9D-2979R | ccAAGCTTGAAGACCACTGGGTGGCTGAC |  |
| S11U-1483F(1) | cgGAATTCCGAAACACGAAGCCCAGCCAGTCC | Left arm of myxovirescin gene cluster |
| S11U-2619R(2) | gcTCTAGAGCAGCATCAGCCCCTTGTAGACGA |  |
| S11D- 141f(3) | gcTCTAGAGCCCACTGAAGGCATCACGGTAAC | Right arm of myxovirescin gene cluster |
| S11D-2093R(4) | ccAAGCTTGCCGCTCCTCGTGCTTGAAGTAGA |  |
| S17U-807F | cgGAATTCGCGATGCTGGCACTGGACCGAGGC | Left arm of myxalamid gene cluster |
| S17U-2945R | gcTCTAGACGGCAGGCGGCGGAAGTAGAGGAT |  |
| S17D-767f | gcTCTAGACGCCATTGGGGACCCGCTGAAGAC | Right arm of myxalamid gene cluster |
| S17D-2695R | ccAAGCTTGGGCGACCTGCTGAAGAACACGGC |  |
| S11U-600F(7) | GCGAACGCCAGCAACACCAACT | Upstream of left arm of myxovirescin cluster |
| S11U-500F(8) | GTGGAGCACGACCGCAACGACA |  |
| S11D-20F(5) | ACCGGGGAACGCTCGGAAATCA | Upstream of of right arm of myxovirescin cluster |
| S11D-97R(9) | GGCAGGGTGAAGGCGATGTTGA | downstream of right arm of myxovirescin cluster |
| S11D-130R(10) | CGCTTGATGACCAGGGTGGCGTAG |  |
| S11D-192R(6) | CGGCAGGGTGAAGGCGATGTTGA |  |
| Scaff-start-F | GTTTTAGAGCTAGAAATAGCAAG | Conventional method to construct sgRNA cassette shown in Figure 2. |
| sg9-2R | GTTCAAAGAGCCGCCTCCCGTGGAGC GGGAAAT |  |
| sg11-2R | CCATCAAGGGCGGCGTCGGCTGGAGCGGGAAAT |  |
| sg17-1R | TCCCCTTCGTCTCATCGCTGTGGAGCGGGAAAT |  |
| PilAF2 | ccgGAATTCTCGTCAGCGTCACTGCCGAATTTTGTC | Change T7A1 promoter to pilA promoter by Golden Gate Assembly |
| Pil-tRF | taGGTCTCACCGCGGGAATAGCTCAGTTGGTAGAGC |  |
| Pil-tRR | cgGGTCTCAGCGGGGGTCCTCAGAGAAGGTTGCAAC |  |
| tRNA-HinR | cccAAGCTTTGGAGCGGGAAATGGGATTC |  |
| gRNA11-1F | taGGTCTCAGGAGTAGAAGCG GTTTTAGAGCTAGAA | sgRNA cassette constructed by BsaI digestion and self-ligation |
| gRNA11-1R | cgGGTCTCACTCCGATTTGCATGGAGCGGGAAATG |  |
| gRNA11-3F | taGGTCTCACGCCCTTGATGGGTTTTAGAGCTAGAA |  |
| gRNA11-3R | cgGGTCTCAGGCGGCGTCGGCTGGAGCGGGAAATG |  |
| gRNA11-4F | taGGTCTCAGAACCGCCCGCCGTTTTAGAGCTAGAA |  |
| gRNA11-4R | cgGGTCTCAGTTCGGACAGGATGGAGCGGGAAATG |  |
| sg17-R4 | GTGTCTCTAGGTGCCTCGTCTGGAGCGGGAAAT | Conventional method to construct sgRNA cassette shown in Figure 2. |
| sg17-R5 | GTAGCGTCCTGGATGAGTGGTGGAGCGGGAAAT |  |
| sg17-R6 | GTGGCAGCCGCAGCCTCGCGTGGAGCGGGAAAT |  |
| sg17-R7 | GTGGTTGGGGATGGGGCGTCTGGAGCGGGAAAT |  |
| sg17-R8 | GTTTGGAGAGCGACCTCTCGTGGAGCGGGAAAT |  |
| sg17-R9 | GTTGGTTCCTCATCATCGCCTGGAGCGGGAAAT |  |
| sg17-R10 | GTGGAAGGCGCAGGAGCAGGTGGAGCGGGAAAT |  |

underlined characters indicate the site of EcoRI, XbaI, HindIII etc. restricted endonuclease,

Indicate the site produced by BsaI digestion in the Golden Gate Assembly.
